# Toward better control of classical swine fever in wild boars: susceptibility of boar-pig hybrids to a recent Japanese isolate and effectiveness of a bait vaccine

**DOI:** 10.1186/s13567-020-00821-w

**Published:** 2020-07-31

**Authors:** Katsuhiko Fukai, Tatsuya Nishi, Manabu Yamada, Mitsutaka Ikezawa

**Affiliations:** 1 Exotic Disease Research Station, National Institute of Animal Health, National Agriculture and Food Research Organization, 6-20-1 Josui-honcho, Kodaira, Tokyo 187-0022 Japan; 2 National Institute of Animal Health, National Agriculture and Food Research Organization, 3-1-5 Kannondai, Tsukuba, Ibaraki 305-0856 Japan

**Keywords:** bait vaccine, classical swine fever virus, experimental infection, pathogenicity, wild boars

## Abstract

We analyzed the pathogenicity of a recent Japanese classical swine fever virus (CSFV) to wild boars via an experimental infection using boar-pig hybrids as an alternative to wild boars. We also investigated the effectiveness of a bait vaccine against the CSFV. Naïve boar-pig hybrids and pigs showed clinical signs such as fever, leucopenia, anorexia and conjunctivitis following the experimental infection. In contrast, the boar-pig hybrids administered the bait vaccine did not show any clinical signs. Our data indicated that boar-pig hybrids and domestic pigs have similar susceptibility to the recent Japanese CSFV. Additionally, the bait vaccine is effective against the CSFV.

## Introduction, methods, and results

Classical swine fever (CSF) is a highly contagious viral disease caused by the CSF virus (CSFV), and is widely distributed around the world. CSFV belongs to the genus *Pestivirus* of the family *Flaviviridae* [1]. CSFV has three genotypes (1, 2 and 3) and several subgenotypes (1.1–1.4, 2.1–2.3, and 3.1–3.4) [2, 3], although a research group proposed recently a new classification schemes for genotypes and subgenotypes of CSFV [4]. Subgenotypes 1.1, 2.1–2.3 and 3.4 are endemic in East and Southeast Asian countries [5, 6]. The virulence of CSFV ranges from highly virulent with almost 100% mortality to avirulent [7, 8], with many genotype 2 strains showing moderate virulence. The severity of clinical signs related to each strain is dependent on the characteristics of the host including age, breed and health [7, 8]. A subgenotype 2.1d isolate that was moderately virulent in China [9, 10] was highly virulent in South Korea in 2016 [An et al., personal communications]. Clinical diagnosis is difficult in farms infected with low virulent strains [11, 12].

In September 2018, a CSF outbreak occurred in Gifu Prefecture, Japan for the first time in 26 years [13]. Fifty-eight cases were identified in domestic pig farms in 8 prefectures until July 2020 [14]. RT-PCR assay of serum/tonsil samples [15] has detected more than 2400 CSFV-infected wild boars in 17 prefectures through the same date [14]. Routine administration of a bait vaccine to wild boars started in March 2019. Regular administration of a live attenuated GPE^−^ vaccine to domestic pigs was started in October 2019.

We analyzed the pathogenicity and horizontal transmissibility of a recent Japanese CSFV isolate in domestic pigs using experimental infection [16]. The progression of recent CSF outbreaks in Japan suggests that wild boars may contribute to spread of the outbreaks. Therefore, understanding the kinetics of the Japanese isolate in wild boars by experimental infection is essential for the establishment of appropriate control measures and diagnostic laboratory assays for outbreaks. Additionally, the bait vaccine strain, C-strain, may be less effective against sublineage 2.1d strains than other subgenotype 2.1 strains [11]. The Japanese isolate, which belongs to subgenotype 2.1d [17], is ideal for evaluating the effectiveness of the bait vaccine against this strain. The objectives of this study were: (1) to evaluate the pathogenicity of the Japanese CSFV isolate to wild boars using boar-pig hybrids as an alternative to wild boars; (2) to compare the pathogenicity of the isolate between hybrids and domestic pigs; and (3) to evaluate the effectiveness of the bait vaccine in hybrids.

We performed all experimental infections using live viruses in a high-containment facility at the National Institute of Animal Health (NIAH). The high-containment facility is compliant with a containment level for group 4 pathogens described in the OIE Manual of Diagnostic Tests and Vaccines for Terrestrial Animals 2019 [18].

The isolate used for the experimental infection was CSFV JPN/27/2019, which was initially isolated from the 11th reported case using CPK cells and propagated twice in the same cells. Animals used for the experimental infection were six 8-week-old boar-pig hybrids (crossbreed Duroc × wild boar × Duroc) and three 8-week-old pigs (crossbreed Landrace × Large White × Duroc). The animals did not have antibodies against pestivirus before the experimental infection. We first administered one dose of the bait vaccine (RIEMSER Schweinepestoralvakzine, Riemser Arzneimittel AG, Greifswald-Insel Riems, Germany) to three hybrids (Hybrids#1–3, Group 1) 14 days before inoculation with JPN/27/2019. Next, we intraorally administered 1 mL of 10^6.5^ 50% tissue culture infectious dose (TCID_50_) of JPN/27/2019 to Group 1, three naïve hybrids (Hybrids#4–6, Group 2) and three naïve pigs (Pigs#7–9, Group 3). Collection of clinical samples (heparinized whole blood, sera, oral and nasal swabs, and feces) was performed daily for the initial 2 weeks, and twice a week thereafter for the following 2 weeks. Preparation of oral and nasal swabs, and feces was performed as described previously [16]. Observation of clinical signs and counting of total leucocyte numbers were conducted for approximately 1 month. Clinical scores were determined to compare the pathogenicity of JPN/27/2019 between the hybrids and pigs according to criteria described previously [19]. When the hybrids and pigs were dormant or were not be able to stand up, we judged that the animals achieved a humane endpoint and euthanized the animals.

Tissue samples for microscopic examination were obtained from the tonsil, liver, spleen, kidney, heart, lung, stomach, small and large intestine, lymph nodes, cerebrum and cerebellum. The tissue samples were fixed in 10% neutral buffered formalin and embedded in paraffin wax using routine procedures. Dewaxed tissue sections were subjected to hematoxylin and eosin, and gram staining. Sections were labeled with a monoclonal antibody specific to CSFV (WH303, APHA SCIENTIFIC, UK) and a rabbit anti-Salmonella O antiserum (NIAH, Tsukuba, Japan) and counterstained with hematoxylin. The Universal Immuno-enzyme Polymer method with a HISTOFINE simple stain Max PO (M) kit (Nichirei, Tokyo, Japan) was used as the detection system for immunohistochemistry. A routine procedure was used to isolate bacteria form the tonsil, mesenteric lymph nodes and spleen using the Pearlcore ES Salmonella Agar II (Eiken Chemical, Tochigi, Japan).

High Pure Viral RNA kit (Roche Diagnostics, Basel, Switzerland) was used to extract viral RNA from clinical samples. Viral genes were amplified using the SuperScript III One-Step RT-PCR System with Platinum Taq DNA Polymerase (Thermo Fisher Scientific, Waltham, MA, USA) and primers 324 and 326 [15]. According to our evaluation, the RT-PCR assay can detect a minimum of 10^0.5^ TCID_50_/mL of the isolate (data not shown). Classical Swine Fever ELISA kit II (JNC Corp., Tokyo, Japan) was used to examine anti-CSFV antibodies in the serum samples [20].

**Figure 1:**
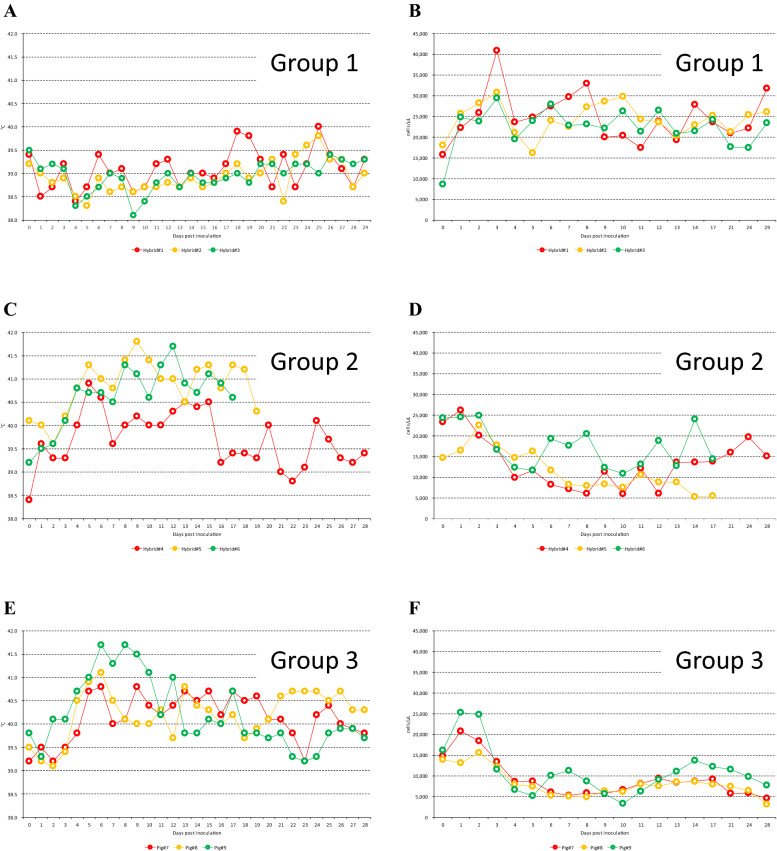
**Body temperature and leucocyte number in hybrids and pigs from Groups 1–3. A**, **C** and **E**: body temperature. **B**, **D** and **F** leucocyte number.

Groups 2 and 3 showed a fever of more than 40 °C from 3–4 and 2–5 days post inoculation (dpi), respectively (Figure 1C, E). The same groups showed leucopenia, with less than 10,000 cells/μL, from 4–7 and 4 dpi, respectively (Figures 1D, F). Hybrid#6 did not show leucopenia during the experimental period (Figure 1D). In contrast, Group 1 did not show fever or leucopenia during the experimental period (Figures 1A, B).

**Figure 2:**
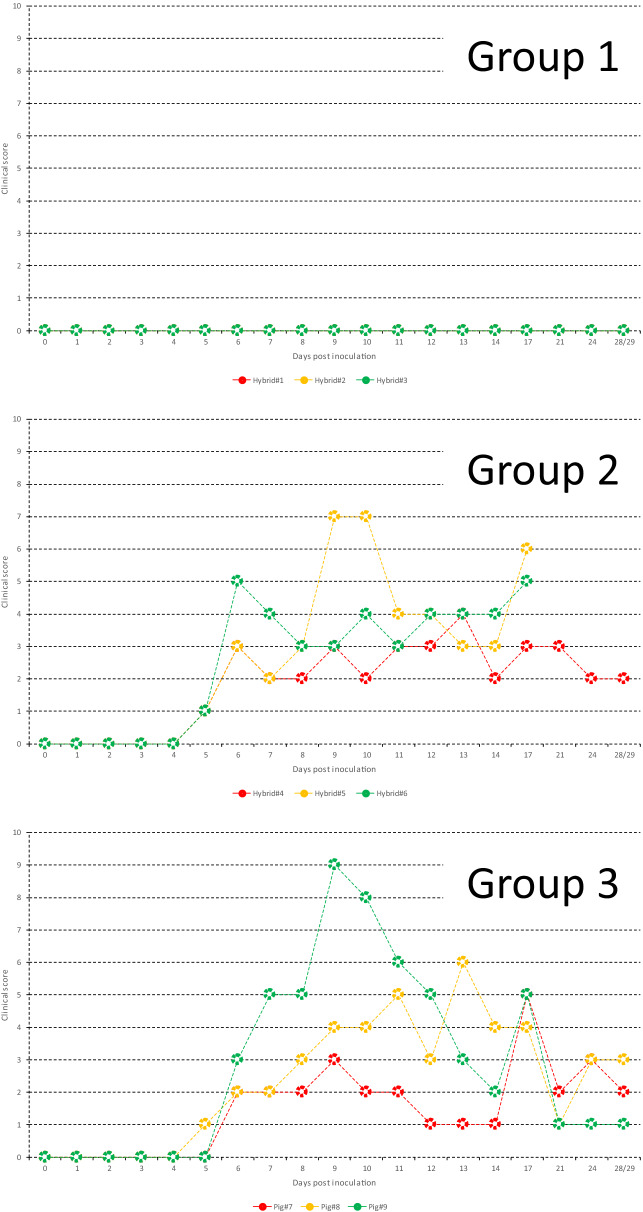
**Clinical score in hybrids and pigs from Groups 1–3.**

Groups 2 and 3 showed anorexia, conjunctivitis, depression, eye mucus, nasal discharge, swaying, shivering, reddened skin, distinct ataxia, cough, diarrhea and bloody feces (Additional file 1). Hybrids#5 and 6 died 19 and 17 dpi, respectively. In contrast, Group 1 did not show any clinical signs. Clinical scores were found at a comparable rate across the experimental period in Groups 2 and 3, but were not found in Group 1 (Figure 2).

Animals in Groups 2 and 3, except for the two dead hybrids, showed the same histological lesions in the examined organs (data not shown). Groups 2 and 3 had immunohistochemically-detected CSFV antigens in all examined organs (data not shown). In contrast, Group 1 did not have histological lesions or CSFV antigens in any examined organs (data not shown).

The dead hybrids had histologically severe suppurative histiocytic bronchopneumonia with thrombus, severe necrotizing enteritis with bacterial colony in the ileum, hemorrhagic lymphadenitis, and hemorrhage with thrombosis in the cerebellum (data not shown). Gram staining showed dissemination of gram-negative bacilli in multisystemic organs (data not shown). While the rabbit anti-Salmonella O antiserum was positive for gram-negative bacilli; the examined organs were negative for the isolation of *Salmonella* spp. The remaining pigs and hybrid in Groups 2 and 3 did not show prominent histological lesions except for hemorrhage and follicular atrophy in the spleen and lymph nodes, and crypt herniation in the intestine (data not shown).

Groups 2 and 3 were positive for viral genes from 3 and 2 dpi, respectively (Table 1). The genes were continuously detected for the remainder of the experimental period. In contrast, Group 1 was negative for viral genes (data not shown). Antibodies were simultaneously detected with viral genes from 10 and 12 dpi in Group 2 and 3, respectively (Table 2).

**Table 1:**
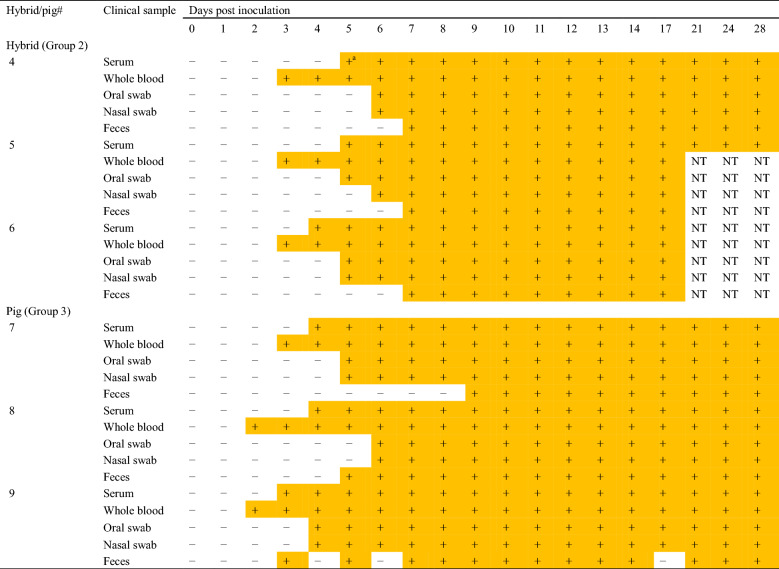
**Detection of viral genes from clinical samples in Groups 2 and 3**

*NT* not tested.

^a^Days when viral genes were detected are colored orange.

**Table 2:**
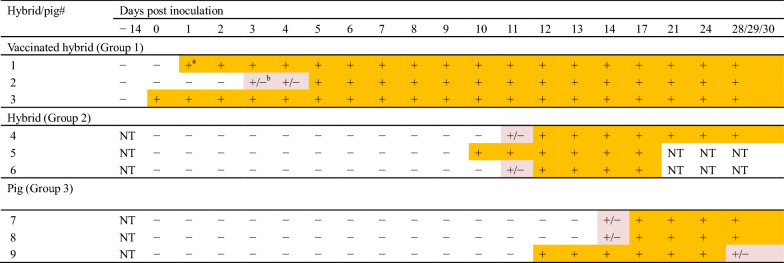
**Detection of antibodies by ELISA in Groups 1–3**

*NT* not tested.

^a^Days when antibodies were detected are colored orange.

^b^Days when results were doubtful are colored light orange.

## Discussion

Groups 2 and 3 showed clinical signs (Additional file 1) while Group 1 did not. Groups 2 and 3 also had histological lesions and CSFV antigens in the examined organs while Group 1 did not. Furthermore, viral genes were detected in Groups 2 and 3 (Table 1) but not in Group 1. Despite evidence suggesting that the bait vaccine strain may be less effective against sublineage 2.1d isolates than other subgenotype 2.1 isolates [11], this study showed that the bait vaccine was effective against the recent Japanese CSFV isolate, which belongs to sublineage 2.1d.

Histological and immunohistochemical examinations suggest that incidental co-infection with *Salmonella* spp. and CSFV may have killed Hybrids#5 and 6 in this study. Immunosuppression followed by CSFV infection may have induced septic conditions associated with salmonellosis in the dead hybrids. Single infection with the CSFV isolate may not have severely affected the other pigs and hybrid in Groups 2 and 3, which did not experience severe salmonellosis before virus inoculation, explaining their survival during the experimental period (Table 2).

Control of CSF has been successful in wild boars in Germany [21]. Administration of the bait vaccine to wild boars started in March 2019 in Japan. However, the number of CSFV-positive wild boars has not been drastically reduced [14]. Significant differences in circumstances exist for wild boars in Germany compared to Japan, including landform, vegetation and food. Therefore, further customization of administration of the bait vaccine is necessary to meet the circumstances of wild boars in Japan.

Domestic pigs and wild boars that were positive for antibodies but negative for viral genes have been identified in the field in Japan [Ministry of Agriculture, Forestry and Fisheries of Japan, unpublished data]. In this study, after the hybrids and pigs were infected with CSFV, viral genes were detected initially in the infected animals. Antibodies were detected subsequently in the animals. Then, the viral genes may disappear earlier than the antibodies in the animals. Namely, results in this study suggest that time course of such domestic pigs and wild boars in the field, which are positive for antibodies but negative for viral genes, is relatively long; therefore, viral contamination may occur in farms and fields for a long periods without detection by the animals.

In conclusion, the findings in this study suggest that: (1) the recent Japanese CSFV isolate shows pathogenicity to boar-pig hybrids; (2) the pathogenicity of the Japanese isolate is similar between boar-pig hybrids and pigs; (3) the bait vaccine can protect against not only clinical and pathological manifestation but also viremia and virus excretion into clinical samples in boar-pig hybrids. Findings from this study are valuable for establishing and improving control measures, diagnostic laboratory assays and guidelines for CSF caused by not only the recent Japanese CSFV isolate but also other CSFV strains.

## Supplementary information

**Additional file 1.** Days post inoculation when clinical signs were observed in Groups 2 and 3.

## Data Availability

Not applicable.

## Abbreviations

CSF: classical swine fever
CSFV: CSF virus
NIAH: National Institute of Animal Health
TCID_50_: 50% tissue culture infectious dose
