# Supplementary material for: Toward better control of classical swine fever in wild boars: susceptibility of boar-pig hybrids to a recent Japanese isolate and effectiveness of a bait vaccine
Source: Vet Res. 2020 Jul 31;51:96. doi: 10.1186/s13567-020-00821-w (PMC7393845; doi:10.1186/s13567-020-00821-w)
Supplement: Supplementary file 1 — Additional file 1. Days post inoculation when clinical signs were observed in Groups 2 and 3. [file 13567_2020_821_MOESM1_ESM.docx]

**Additional file 1 Days post inoculation when clinical signs were observed in Groups 2 and 3**

| **Clinical sign** | **Hybrid/pig#** | | | | | |
| --- | --- | --- | --- | --- | --- | --- |
|  | **Hybrid (Group 2)** | | | **Pig (Group 3)** | | |
|  | **4** | **5** | **6** | **7** | **8** | **9** |
| Anorexia | 5–14, 17, 21, 24 | 5–14, 17 | 5–14, 17 | 6–14, 17, 21, 24 | 6–14, 17, 21, 24 | 6–14, 17, 21 |
| Conjunctivitis | 8–10, 12, 13 | 9, 10 | 6, 7, 9, 10, 12 | 6–11, 17 | 5–14, 17 | 7–11, 17 |
| Depression | 6, 11 | 6 | 6, 11, 14 | -^a^ | 11, 13 | 7, 9, 10 |
| Eye mucus | 9–14, 17, 21, 24 | 8–14, 17 | 6–14, 17 | - | 8–14, 24 | 6–13 |
| Nasal discharge | 13 | 8–14, 17 | 8, 10, 12, 13 | - | - | 7–10 |
| Swaying | - | 9, 10, 17 | 17 | - | - | 8–12 |
| Shivering | - | 9, 10 | - | 17 | 10, 11, 13, 14, 17 | 9–12, 17 |
| Reddened skin | 17, 21 | 17 | 13, 14, 17 | 17, 21, 24 | 13, 17, 24 | 17 |
| Distinct ataxia | - | 17 | 17 | - | - | - |
| Cough | - | - | - | 17 | - | 6 |
| Diarrhea | - | - | - | - | - | 10–14, 17 |
| Bloody feces | - | - | - | - | 24 | - |
| Death | - | 19 | 17 | - | - | - |

^a^ Clinical signs not observed during the experimental period.
